# The effects of cages implantation on surgical and adjacent segmental intervertebral foramina

**DOI:** 10.1186/s13018-021-02421-6

**Published:** 2021-04-27

**Authors:** Changyan Wu, Xiaojuan Yang, Xu Gao, Liwei Shao, Fang Li, Yunxin Sun, Xiaoyu Liu, Shuaihao Yao, Yifu Sun

**Affiliations:** 1 Department of Spine Surgery, China-Japan Union Hospital of Jilin University, 126 Xiantai St., Erdao District, Changchun City, People’s Republic of China; 2 Medical Imaging Department, China-Japan Union Hospital of Jilin University, Changchun City, People’s Republic of China

**Keywords:** Intervertebral foramen, Anterior cervical discectomy and fusion, Distraction, Cage selection

## Abstract

**Objection:**

The overarching goal of our research was to compare the clinical and radiological outcomes with different sizes of cages implantation in anterior cervical discectomy and fusion (ACDF), and to evaluate the effects on surgical and adjacent segmental intervertebral foramina.

**Methods:**

The clinical data of 61 patients were analyzed retrospectively. The radiological data included the surgical intervertebral disk space height before (*H*_0_) and after surgery (*H*), the preoperative mean height of adjacent segments (*H*_m_), the area and height of the surgical and adjacent segment foramen, the surgical segmental Cobb angle (*α*_1_), and C2-7Cobb angle (*α*_2_). The calculation of clinical data was conducted by Japanese Orthopaedic Association Scores (JOA), the recovery rate of JOA scores and visual analog scales (VAS). In accordance with the different ranges of distraction (*H*/*H*_m_), patients were classified into three groups: group A (*H*/*H*_m_<1.20, *n*=13), group B (1.20≤*H*/*H*_m_≤1.80, *n*=37), and group C (*H*/*H*_m_>1.80, *n*=11).

**Results:**

After the operation and at the final follow-up, our data has demonstrated that the area and height of surgical segmental foramen all increased by comparing those of preoperation in three groups (all *P*<0.05). However, except for a decrease in group C (all *P*<0.05), the adjacent segmental foramina showed no significant changes (all *P*>0.05). The area and height of the surgical segment foramen and the distraction degree were positively correlated (0<*R*<1, all *P*<0.05), while the adjacent segments were negatively correlated with it (0<*R*<1, *P*=0.002~0.067). JOA scores improved markedly in all groups with similar recovery rates. However, during the final follow-up (*P*=0.034), it was observed that there were significant differences in visual simulation scores among the three groups.

**Conclusion:**

The oversize cage might give a rise to a negative impact on the adjacent intervertebral foramen in ACDF. The mean value of the adjacent intervertebral disk space height (*H*_m_) could be used as a reference standard. Moreover, the 1.20~1.80 fold of distraction (*H*/*H*_m_) with optimal cages would achieve a better long-term prognosis.

## Introduction

Anterior cervical discectomy and fusion (ACDF) was originally described around the 1950s. Initially, ACDF was performed using the autograft following the experience of Smith, Robinson, and Cloward [1, 2]. Subsequently, a surging number of studies revealed that some severe complications of the donor site might be developed by the autologous bone graft. These complications included wound hematoma and infection, acute or chronic pain, and injury of the lateral femoral cutaneous nerve and so on, with a rate up to 25.3% [3]. On the other hand, bone resorption, the collapse of intervertebral disk space, and so on are all examples of complications related to the acceptor site. Some researchers, therefore, attempted to explore an alternative to the autograft. Afterwards, some materials were designed to assist fusion emerged, comprising allografts, titanium cage, carbon fiber cages, and polyetheretherketone (PEEK) cages, etc. At the beginning of this century, Yang et al. found that both of the PEEK cage and the autogenous iliac crest graft would expand the area of the foramen. However, the postoperative height of the foramen only shows a pattern of growth within the group using the PEEK cage [4].

In 1989, the application of the Caspar plating system effectively optimized the cage implantation and strengthened the fixation of anterior screw and plate fixation [5]. Nowadays, with a series of advantages discovered, like immediate stability, effective intervertebral distraction and adequate neural decompression, ACDF became the gold standard for the surgical treatment of cervical spondylosis [6, 7].

The main purpose of ACDF is neural decompression, which is a possible indirect result of the intervertebral distraction and cage insertion. Previous studies reported that the area and height of the surgical segment foramen would increase after implanting the cage [4, 8, 9]. Unfortunately, there is remaining an absence of information about the foramen of adjacent segments since only a limited number of studies specialized in it. Therefore, our study aimed to compare the clinical and radiological outcomes of inserting different sizes of cages in ACDF and evaluate the effects for surgical and adjacent segment foramen.

## Methods

### Patient sample

The present retrospective study involved 61 patients who underwent single-level ACDF in our hospital from February 2017 to June 2020. The follow-up period was at least 6 months. All the data were obtained independently by three researchers and the mean values of multiple measurements were used in subsequent analyses.

### Patient selection

Inclusion criteria: (1) the patients were diagnosed as degenerative cervical spondylosis which was confirmed by preoperative magnetic resonance imaging (MRI), computed tomography (CT), X-ray, and physical examination; (2) single-segment ACDF at the level of C3~C6; (3) the PEEK cage was the product of the same manufacturer (Paonan Biotech Co., Ltd., Taiwan); (4) there was CT scans and plain radiographs of the cervical spine after the operation and at the last follow-up; and (5) patients were followed up for more than 6 months.

Exclusion criteria: (1) patients with malignant tumors, severe infections, or ossification of the posterior longitudinal ligament of the cervical spine; (2) patients with anterior and posterior cervical surgery; (3) patients with cervical spine fractures or dislocations; (4) patients with 2 or more segmental lesions; and (5) no history of cervical surgery.

### Surgical interventions

All the patients were operated by the senior surgeons in our hospital. The left-side anterolateral approach was used to fully expose the platysma and deep cervical fascia. The vascular sheath was drawn to the outside. The trachea and esophagus were drawn to the inside, reaching the front of the cervical vertebral body. After cutting the anterior fascia, the target intervertebral disk was displayed, and a positioning needle was inserted to confirm the correct segment. Subsequently, a #15 blade scalpel was used to remove the corresponding anulus fibrosis, and pituitary rongeur was used to remove the intervertebral disk tissue. After the Caspar nail was implanted into the adjacent vertebral bodies, the intervertebral space was opened by Caspar distractor. The pituitary rongeur and micro curettes were alternately used to remove the compressive materials such as herniated disk and posterior longitudinal ligament. After determining the optimal size of a trial cage, an appropriate-sized cage (Paonan Biotech Co., Ltd., Taiwan) filled with allograft bone was placed into the intervertebral disk space of the target segment. Then the Caspar distractor was released. After confirming the stability of the cage, the anterior plate system was applied (Beijing Libeier Bio-Engineering Institute Co., Ltd., Beijing). Postoperatively, all patients were required to wear a cervical collar for 4~6 weeks.

### Clinical evaluation

The JOA score was recorded from preoperation to the final follow-up, with the scale from 0 to 17 means from the worst to best, respectively. The recovery rate of JOA scores represented the degree of surgical efficacy after operation, and the following formula established by Hirabayashi was to calculate the rate [10]:

$$ \mathrm{Recovery}\ \mathrm{rate}=\frac{\left(\mathrm{postoperative}\ \mathrm{JOA}\ \mathrm{score}\kern0.5em -\kern0.5em \mathrm{preoperative}\ \mathrm{JOA}\ \mathrm{score}\right)}{\left(17-\kern0.5em \mathrm{preoperative}\ \mathrm{JOA}\ \mathrm{score}\right)}\times 100\% $$

The VAS score ranging from 0 to 10, was to evaluate neck pain, preoperatively, postoperatively, as well as at the last follow-up.

### Radiographic measurement

**Fig. 1:**
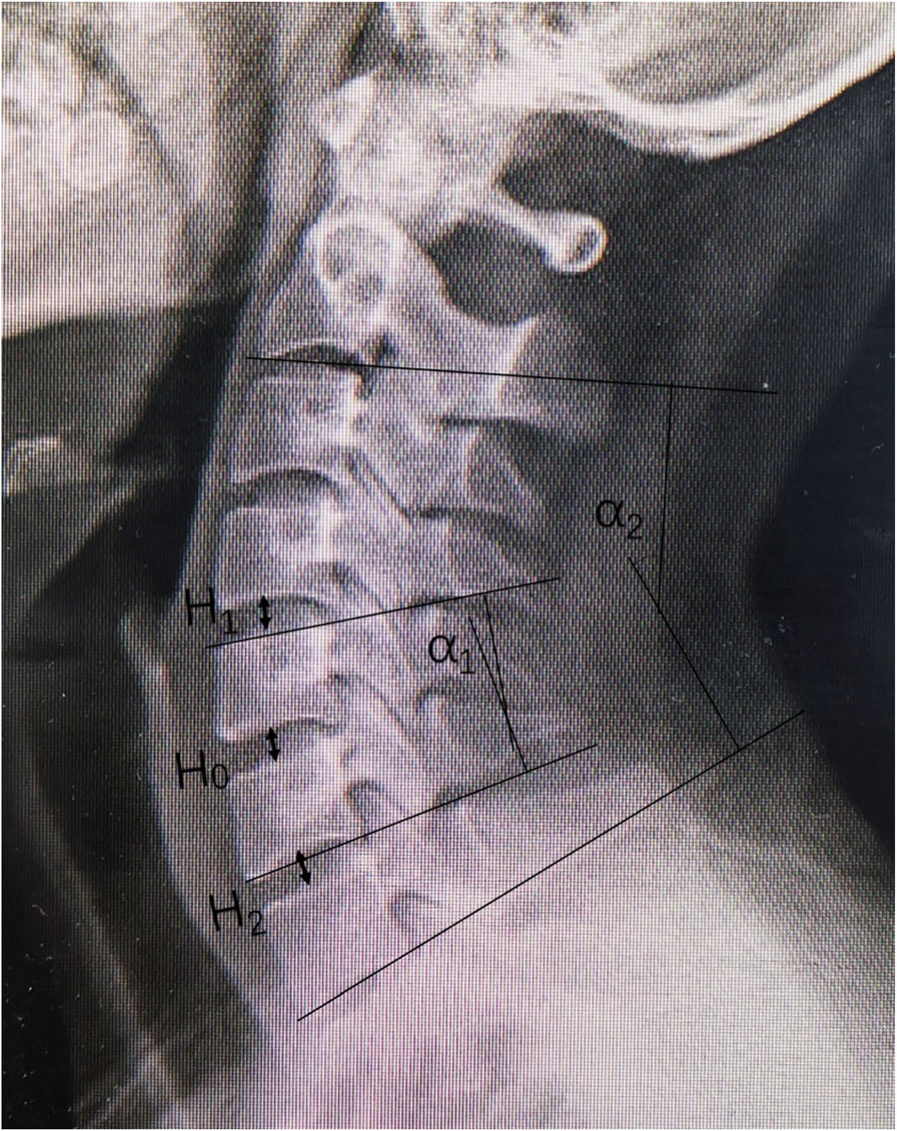
Measurement of intervertebral space and cervical curvature. *H*_0_, the preoperative height of surgical segment intervertebral disk space, the distance measured from the middle of an inferior endplate of the upper vertebral body to the superior endplate of the lower vertebral body. *H*_1_, upper intervertebral disk space height before the operation. *H*_2_, inferior intervertebral disk space height before the operation. *α*_1_, surgical segmental curvature, angle between two lines perpendicular to the superior endplate of the upper vertebral body and the inferior endplate of the lower vertebral body in the surgical segment. *α*_2_, global curvature, angle between two lines perpendicular to the inferior endplate of the C2 and the inferior endplate of the C7

The measured distance was the intervertebral disk space from the middle of an inferior endplate of the upper vertebral body to the superior endplate of the lower vertebral body on the cervical lateral X-ray plain film [11]. The surgical segment height was measured before (*H*_0_) and after surgery (*H*). Meanwhile, the intervertebral disk space height of the upper (*H*_1_) and lower (*H*_2_) segments were measured before surgery. Also, the calculation of Δ*H* of *H*_1_, and *H*_2_ was done after surgery (ΔSH_1_=*H*_1_−postoperative *H*_1_; ΔSH_2_=*H*_2_−postoperative *H*_2_) and at the last follow-up (ΔFH_1_=*H*_1_−follow-up *H*_1_; ΔFH_2_=*H*_2_−follow-up *H*_2_). The mean value of *H*_1_ and *H*_2_ was calculated as the mean intervertebral disk space height of the surgical segment (*H*_m_=(*H*_1_+*H*_2_)/2) (Fig. 1).

The cervical curvature, including regional curvature (*α*_1_) and global curvature (*α*_2_), were measured by the method of Cobb. On this basis, regional curvature (*α*_1_) was defined as the angle between two lines perpendicular to the superior endplate of the upper vertebral body and the inferior endplate of the lower vertebral body in the surgical segment. Global curvature (*α*_2_) was defined as the angle between two lines perpendicular to the inferior endplate of the C2 and the inferior endplate of the C7. Both *α*_1_ and *α*_2_ were measured on the neutral lateral X-ray plain film (Fig. 1).

**Fig. 2:**
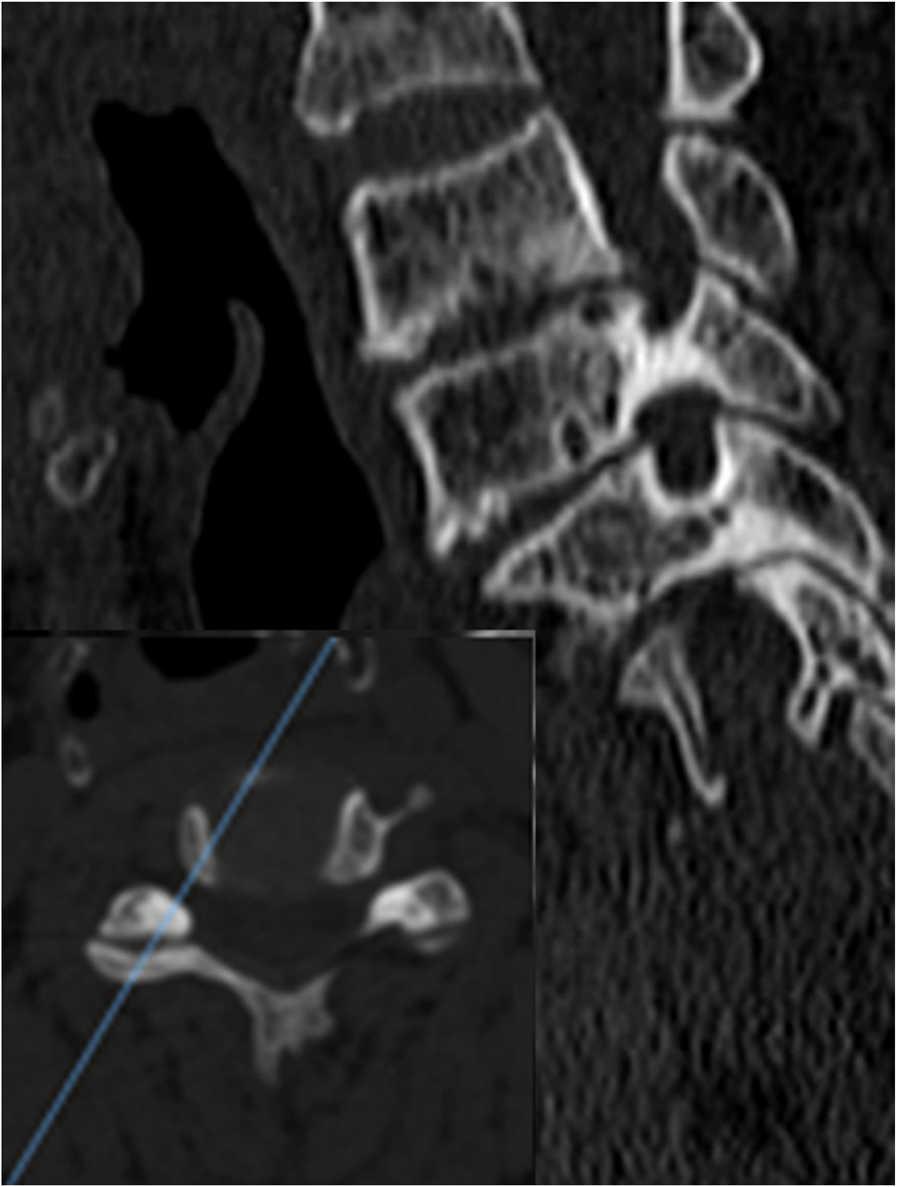
Oblique sagittal reconstruction of the intervertebral foramen

**Fig. 3:**
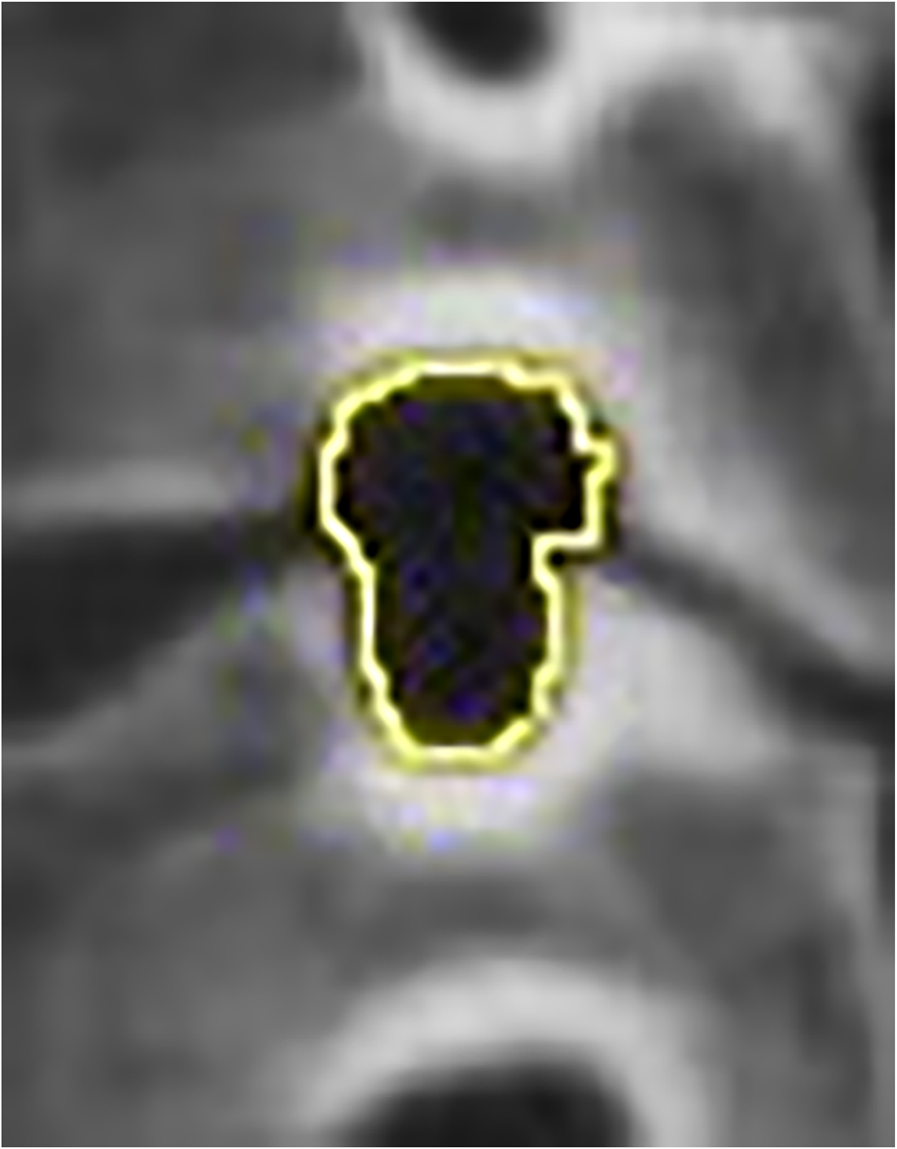
Measurement of intervertebral foramen area by image J

**Fig. 4:**
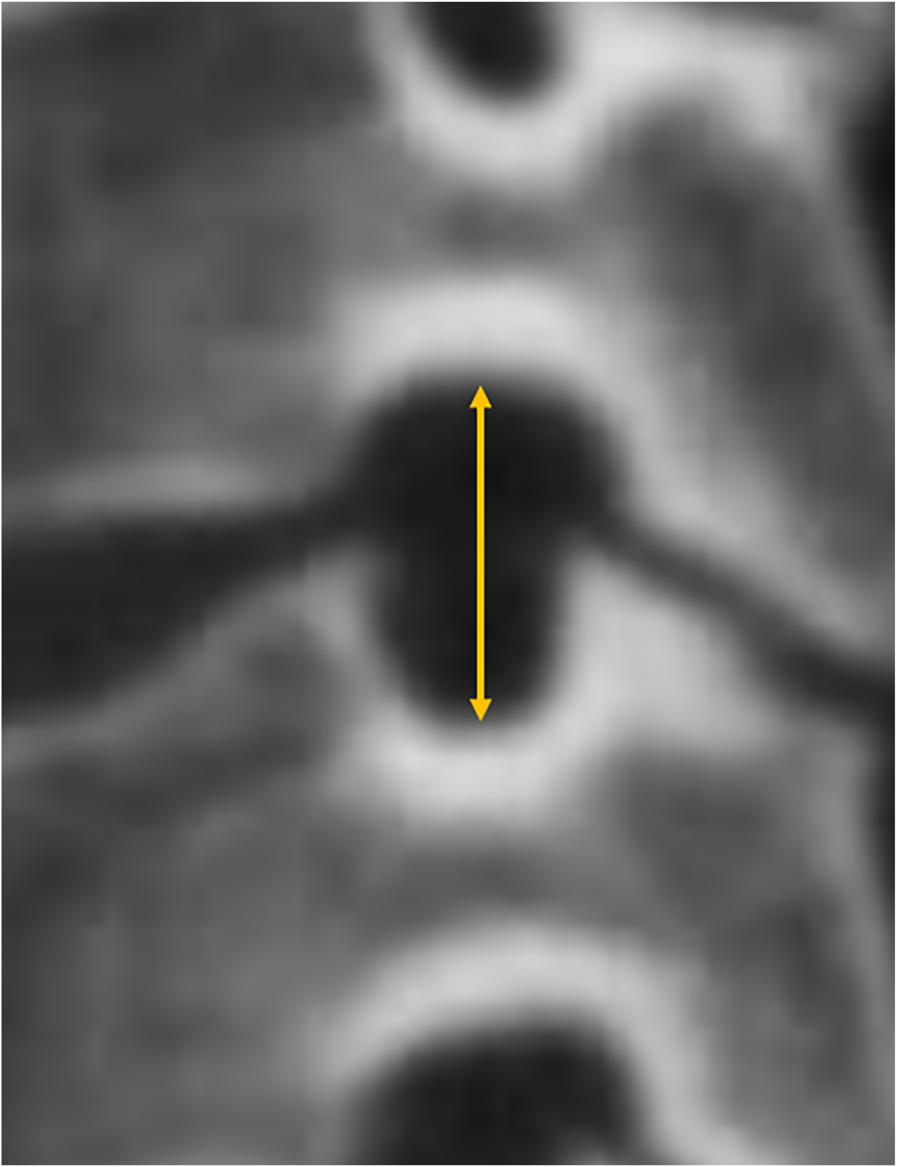
Measurement of intervertebral foramen height, which is the distance between the midpoints of the upper and lower pedicles

CT scans had been carried out since the operation (within 3 days) to the last follow-up (at least 6 months). We selected the image of the intervertebral foramen plane of the corresponding segment, took the intervertebral space plane parallel to the position of the endplate, before adjusting it to the plane of the largest intervertebral foramen. Oblique sagittal reconstruction (surgical and adjacent segment, right and left foramen) of every intervertebral foramen (perpendicular to the axis of the intervertebral foramen) was under the bone window [12] (Fig. 2). Then, the reconstructed foramen pictures were imported into the software of Image J (National Institutes of Health, USA). After setting the scale, the mouse was manually dragged to measure the foramen area. Meanwhile, the measurement of the height for the foramen was the distance between the midpoints of the upper and lower pedicles (Figs. 3 and 4).

### Statistical analysis

All the statistical analyses were conducted by using the SPSS software version 25.0 (International Business Machines Corporation, USA). The data were described as mean ± standard deviation (*x* ± *s*), and the Shapiro-Wilk test or the normal Q-Q plot were used to verify its normal distribution. Intra-group differences were calculated by a paired *t* test. One-way analysis of variance (ANOVA) was used to compare the differences among groups. Post-hoc tests were conducted using the Bonferroni method. Nonparametric test will be used for data with non-normal distribution. The correlation analyses were implemented in Pearson’s or Spearman’s tests. It was considered to be of statistical significance with *P*<0.05. The intraclass correlation coefficient (ICC) was used to evaluate the interobserver reliability (>0.75, excellent; <0.40, poor).

## Results

A total of 61 patients were included in this study. After expressing the intervertebral distraction degree (*H*/*H*_m_) of all patients on the coordinate axis, we found that there were three different segments with the boundary of 1.20, 1.80. Additionally, with previous studies available for consultation, we derived the intervertebral tension degree ranged from 1.20 to 2.00 [1, 9, 13–17]. Therefore, we divided the patients into three groups: group A, *H*/*H*_m_<1.20; group B, 1.20≤ *H*/*H*_m_≤1.80; and group C, *H*/*H*_m_>1.80. There were 13 patients in group A, 37 patients in group B, and 11 patients in group C. The baseline clinical characteristics among the three groups showed no significant difference in terms of gender (*P*=0.431), age (*P*=0.956), and operated levels (*P*=0.424) (Table 1). Apart from the above, the ICC was excellent (all>0.75).

**Table 1:** General characteristics of patients

	Group A	Group B	Group C	*P* value
No. patients	13	37	11	
Age (years)	52.000±10.384	51.108±10.244	51.818±10.962	0.956
Gender (male/female)	10/3	27/10	6/5	0.431
Surgical segment				
C3/4	4	5	2	0.424
C4/5	0	6	4	
C5/6	9	26	5	
Intervertebral space height (mm)				
*H*_m_	7.068±1.198	6.881±1.121	7.296±1.237	0.564
*H*_0_	6.613±1.901	6.625±1.324	7.593±1.401	0.151
*P*_*0*_ value (*H*_m_ vs *H*_0_)				0.167

*H*_m_ mean intervertebral disk space height of the adjacent upper and lower segments before the operation, *H*_0_ preoperative surgical intervertebral disk space height

### Clinical outcomes

During the postoperative and last follow-up period, the JOA scores in all three groups markedly improved (all *P*<0.05), and the VAS scores decreased simultaneously (all *P*<0.05). No significant difference was found for JOA scores and the recovery rate among the three groups (all *P*>0.05). As for the VAS score, there was no difference among the three groups throughout the operation (all *P*>0.05). However, at the last follow-up, there was a lower score in group B (*P*=0.034) (Table 2).

**Table 2:** Clinical outcomes

	Group A	Group B	Group C	*P* value
JOA scores				
Preoperation	9.539±1.266	9.487±1.677	9.455±1.440	0.957
Postoperation	13.231±1.739*	13.189±2.196*	12.909±2.700*	1.000
Follow-up	13.231±1.878*	13.243±1.571*	12.545±1.753*	0.301
JOA recovery rate (%)				
Preoperation	51.200±15.464	51.193±23.040	49.350±25.398	0.981
Postoperation	50.690±18.321	50.243±17.529	43.035±15.395	0.186
VAS scores				
Preoperation	5.231±1.641	4.973±1.756	5.273±1.679	0.861
Postoperation	3.000±1.225*	2.730±1.170*	2.909±1.221*	0.804
Follow-up	2.615±0.768*^B^	1.919±1.064*	2.727±1.104*^B^	0.034

*P < 0.05 compared with preoperative value

^B^*P* < 0.05 compared with group B

JOA scores, Japanese Orthopaedic Association Scores, on a scale from 0 to 17

VAS scores, visual analog scales, on a scale from 0 to 10

### Radiological feature

When it comes to *H*_m_ and *H*_0_, no significant difference was found among the three groups before the operation (*P*=0.564, *P*=0.151). The statistical difference between *H*_0_ and *H*_m_ in all over of patients was not found (*P*=0.167) (Table 1). What is more, although there was no significant difference in Δ*H* among the three groups, Δ*H* of group C was larger than that of other two groups in both postoperative and follow-up (all *P*>0.05) (Table 3).

**Table 3:** The reduction of intervertebral space height at the adjacent segment

	Group A	Group B	Group C	*P* value
ΔSH_1_ (mm)	0.232±0.588	0.107±0.789	0.398±1.033	0.560
ΔSH_2_ (mm)	0.261±0.894	0.208±0.850	0.322±1.011	0.928
ΔFH_1_ (mm)	0.471±1.122	0.329±0.897	0.800±1.031	0.391
ΔFH_2_ (mm)	0.511±1.024	0.492±0.929	0.833±1.142	0.596

ΔSH_1_, the reduction of intervertebral disk space height at the adjacent upper segment after surgery (ΔSH_1_=*H*_1_−postoperative H_1_); ΔSH_2_, the reduction of intervertebral disk space height at the adjacent lower segment after surgery (ΔSH_2_=*H*_2_−postoperative H_2_); ΔFH_1_, the reduction of intervertebral disk space height of the adjacent upper segment at the last follow-up (ΔFH_1_=*H*_1_−follow-up *H*); ΔFH2, the reduction of intervertebral disk space height of the adjacent lower segment at the last follow-up (ΔFH_2_=*H*_2_−follow-up *H*_2_)

Since no difference was found between the left and right foramen in all three groups (all *P*>0.05), the left and right foramen were merged for analysis. During the postoperative and follow-up period, the area and height of the surgical segment foramen within the three groups all increased significantly comparing with the preoperative parameters (all *P*<0.05). Regarding the foramen of the upper and lower segments, there is no significant difference in group A and B after the operation and at last follow-up (all *P*>0.05), whereas observing a statistical decrease in group C (all *P*<0.05) (Table 4).

**Table 4:** Changes of foramen area and height

	Group A	Group B	Group C	*P* value
Area (mm^2^)				
Upper segment				
Preoperation	53.403±11.541	49.953±11.332	45.672±9.784	0.060
Postoperation	55.927±14.731^C^	49.251±9.814^C^	41.525±7.744*	0.010
Follow-up	53.349±13.903^C^	49.011±9.679^C^	39.938±7.962*	<0.001
Surgical segment				
Preoperation	35.643±15.483	34.851±8.590	34.832±10.251	0.945
Postoperation	39.847±11.617*	42.105±8.527*	46.314±9.700*	0.061
Follow-up	39.755±10.802*	41.450±8.690*	45.631±9.164*	0.081
Lower segment				
Preoperation	43.509±9.122	46.213±9.564	41.384±10.808	0.097
Postoperation	42.266±9.062	45.651±8.132^C^	37.288±9.449*	<0.001
Follow-up	42.281±8.654	45.074±7.912^C^	36.477±8.830*	<0.001
Height (mm)				
Upper segment				
Preoperation	8.683±1.032	8.674±1.355	8.014±0.832	0.072
Postoperation	8.748±0.969^C^	8.617±1.315^C^	7.379±0.856*	<0.001
Follow-up	8.633±0.882^C^	8.555±1.271^C^	7.243±0.812*	<0.001
Surgical segment				
Preoperation	7.252±1.524	7.382±1.029	7.106±0.925	0.586
Postoperation	8.110±1.254*	8.452±0.973*	8.725±0.995*	0.124
Follow-up	8.033±1.165*	8.374±0.954*	8.665±0.998*	0.098
Lower segment				
Preoperation	7.669±1.463	8.148±1.194	7.565±1.279	0.083
Postoperation	7.921±1.178	8.218±1.169^C^	7.154±1.271*	0.002
Follow-up	7.824±1.085^C^	8.067±0.940^C^	6.960±0.957*	<0.001

*P < 0.05 compared with preoperative value

^C^*P* < 0.05 compared with group C

Through the correlation analysis, the area and height of the surgical segment foramen and the degree of intervertebral distraction (*H*/*H*_m_) were positively correlated (0<*R*<1, all *P*<0.05). However, the adjacent segments and *H*/*H*_m_ were negatively correlated. Except the postoperative area (*R*=−0.243, *P*=0.059) and height (*R*=−0.236, *P*=0.067) of the upper segment showed no statistically significant correlations with *H*/*H*_m_, there were statistically significant correlations between *H*/*H*_m_ and the upper segment at follow-up (area: *R*=−0.350, *P*=0.006; height: *R*=−0.296, *P*=0.020), lower segment after the operation (area: *R*=−0.259, *P*=0.044; height: *R*=−0.262, *P*=0.041) and at follow-up (area: *R*=−0.396, *P*=0.002; height: *R*=−0.276, *P*=0.031) (Table 5).

**Table 5:** Correlation of the area and height of the intervertebral foramen and the degree of intervertebral distraction

	Surgical segment		Upper segment		Lower segment	
	Postoperation	Follow-up	Postoperation	Follow-up	Postoperation	Follow-up
Area (mm^2^)						
*R*	0.432	0.372	−0.243	−0.350	−0.259	−0.396
*P*	0.001	0.003	0.059	0.006	0.044	0.002
Height (mm^2^)						
*R*	0.379	0.353	−0.236	−0.296	−0.262	−0.276
*P*	0.003	0.005	0.067	0.020	0.041	0.031

*R* correlation coefficient, *R* > 0 indicates positive correlation, *R* < 0 indicates negative correlation

The *α*_2_ in groups A, B, and C showed no significant difference after the operation and during the follow-up (all *P*>0.05). However, the *α*_1_ showed obvious improvement after the operation and at the last follow-up, especially in group C (all *P*<0.05). Post hoc tests indicated that the improvement of group C was more remarkable than groups A and B (Table 6).

**Table 6:** Changes in cervical curvature

	Group A	Group B	Group C	*P* value
α_1_ (°)				
Preoperation	2.875±5.989	3.241±4.449	3.687±1.388	0.907
Postoperation	6.105±4.132*^C^	7.136±4.223*^C^	10.348±2.859*	0.030
Follow-up	6.256±4.072*	6.583±4.202*	9.418±2.150*	0.086
α_2_ (°)				
Preoperation	11.774±11.626	11.376±10.116	12.940±5.267	0.898
Postoperation	11.431±12.466	13.201±9.081	15.626±4.169	0.547
Follow-up	11.064±10.262	11.524±8.672	15.985±4.142	0.27
Increase of α_1_ (°)				
Postoperation	3.153±2.910^C^	3.911±2.696^C^	6.661±2.921	0.007
Follow-up	3.288±2.951	3.372±2.519^C^	5.730±2.428	0.028

*P < 0.05 compared with preoperative value

^C^*P* < 0.05 compared with group C

*α*_1_, Surgical Cobb angle

*α*_2_, C2-7Cobb angle

Postoperative increase of *α*_1_, postoperative α_1_−preoperation *α*_1_

Follow-up increase of *α*_1_, follow-up *α*_1_−follow-up *α*_1_

## Discussion

Degenerative spinal disease is a focal public health issue, degenerative cervical disk disease in particular, which occupied around 20~30% of patients with pain in the vertebral column and was expected to increase shortly [18]. Presently, with the advanced technique of cage implantation and plate internal fixation, ACDF has become the gold standard for the treatment of cervical spondylosis [6, 7]. In our study, all patients have applied the cage filled with allograft bone, and anterior plate fixation. It turned out that ACDF was able to achieve the recovery of intervertebral space height and the maintenance of cervical stability. With the application of allograft bone, the time for hospital stays was reduced and donor site-related complications due to autologous bone grafting were avoided as well [19]. In addition, compared with autologous iliac crest grafts and titanium cages, the PEEK cages reduced the possibility of graft fracture and intervertebral collapse [20]. Moreover, the rate of fusion would be enhanced considerably by using allograft bone and plate fixation [21, 22]. Meanwhile, cages with various models could meet the needs of almost all patients.

Notably, the degree of distraction in ACDF remained controversial. Smith and Robinson considered the height of bone graft as10~15 mm [1]. While White et al. commended that the appropriate height was 4~5 mm [16]. Subsequently, Xiong et al. confirmed that the 0.50~1.00 fold intervertebral distraction was good [17]. In Albert’s research, he found the increase of the foramen area and height in ACDF [8], which was discredited by Nguyen [23], subsequently. In addition, previous studies showed that when the height of the bone graft was increased to a certain point, the size of the foramen would begin to decrease [9]. However, little attention was paid to the adjacent segment foramen. Therefore, the purpose of this study was to evaluate the effect on surgical and adjacent segment foramen such as area and height after implanting different sizes of cages. The width of the foramen was not measured due to the change that could not be attributed to the implantation of a cage.

Previous studies were confusing because most of them took the preoperative height of the surgical segment intervertebral disk space as a reference standard. We have considered certain situation where the individual difference and the extremum of the surgical segment height because of the different disease progression were considered. Therefore, we used the mean value of the adjacent intervertebral disk space height (*H*_m_) as the reference standard. Consequently, no significant difference between *H*_0_ and *H*_m_ was uncovered (*P*=0.167). The reason might be the loss of intervertebral disk space height being an overall cooperation mechanism in the cervical spine [11]. The reference standard applied in our study not only could represent the intervertebral disk space height of the surgical segment but also minimize the influence of outliers.

Significant improvement with the height and area of the intervertebral foramen in the surgical segment was approved in three groups after the operation and at the last follow-up (all *P*<0.05). And the area and height of the surgical segment foramen and the degree of intervertebral distraction (*H*/*H*_m_) were positively correlated (0<*R*<1, all *P*<0.05). At the surgical level, the improvement of the foraminal area and height might be the result of opening the intervertebral space after inserting cages. Our research was consistent with the observation from previous studies, indicating that cage implantation contributed to increasing the intervertebral foraminal area and height of the surgical segment [4, 8, 9]. This situation might indirectly lead to the decompression of the spinal cord and nerve root. As for the adjacent segment, however, only group C showed the statistical decrease of the height and area after the operation and at the final follow-up (*P*<0.05). On the other hand, the adjacent segments and *H*/*H*_m_ were negatively correlated. The diminutions of the foraminal area and height at the adjacent levels might be the result that an oversized cage would contribute to over distraction of intervertebral space. On the basis of our conjecture, we further considered that abnormal stress might increase, as well as the intervertebral disk space height, the foraminal height and area in the adjacent segments might decrease. Our data demonstrated that the height of adjacent intervertebral space decreased in all three groups, especially in group C, which seems to coincide with our inference.

Yang et al. found that with the increase of the intervertebral space distraction, the size of the intervertebral foramen began to decrease until a certain point. And, they concluded that 160% of the mean height of adjacent intervertebral spaces was the optimal degree of distraction [9]. In our study, 1.20~1.80 fold distraction was the optimal range. Nevertheless, we did not observe the decrease of the intervertebral foramen in the surgical segment, which may result from the small clinical samples and large manual measurement error.

After the operation and at the last follow-up, all patients revealed significant improvements in the JOA scores (all *P*<0.005) and diminutions in VAS scores (all *P*<0.05), while the only statistical differences among the three groups at the follow-up (*P*=0.034) was illustrated in the VAS score. Post hoc multiple comparisons identified that the VAS scores of group B were lower in group B than group A (*P*< 0.042) and C (*P*=0.041). Moreover, our result suggested that group C showed higher VAS scores than group B at the final follow-up, which could be explained by the following reasons [24]. Firstly, the increased stress in the disk and facet joints at the adjacent segments caused by the oversized cage, leading to the diminution of the foramen and the structural disorders of facet joints, etc. Secondly, a longstanding high tension of the facet joint capsule and other soft tissues, caused by the oversized cage, might further result in the injury of the joint capsule and ligament even subluxation or dislocation of the facet joints. Besides, the mechanical stimulation on the intervertebral ligament and sinus nerve in the joint capsule, and the distraction stimulus of nerve roots would result in corresponding symptoms of pain. As for group A, the reason for the lower decreases in VAS scores might be the insufficient decompression due to the limited intervertebral distraction, and the excessive stress or disorders of the facet joints [17, 24].

After the operation and at follow-up, the changes of *α*_1_ and *α*_2_ suggested that cages implantation could increase segmental lordosis of each surgical segment without affecting the overall curvature of the cervical spine, after the operation and at follow-up. Prior empirical studies indicated that, ACDF could recover or improve the segmental lordosis at the surgical level and avoid the kyphosis, compared with the posterior approach and simple intervertebral bone grafting. It is generally accepted that the recovery of segmental lordosis and the correction of kyphosis was helpful to relieve clinical symptoms [25, 26], but excessive lordosis might be worse. Kim et al. found that patients with C5 nerve root palsy after the cervical operation had greater lordosis than those without palsy. It might be related to the iatrogenic foramen stenosis caused by the excessive anterior distraction and the sudden rise of the posterior pressure [27]. In accordance with “bowstring principle,” with the increase of lordosis, the spinal cord is more likely to “drift backward” with the increase of lordosis, so that the tension on the nerve roots was significantly increased. In the present study, the differences in changes of segmental lordosis among the three groups were confirmed. Compared with group A (*P*=0.011, *P*=0.083) and group B (*P*=0.016, *P*=0.030), the increase was obvious in group C, compared with group A (*P*=0.011, *P*=0.083) and group B (*P*=0.016, *P*=0.030) in the period of postoperation and follow-up. As mentioned earlier, the VAS score of group C was higher than group B during follow-up. Whether the higher VAS score is related to the excessive segmental lordosis still remain to be explored.

### Shortcomings and prospects

There are limiting factors in our study in terms of the small sample size, manual measurements, and a short follow-up period. In addition, all patients were required to wear a cervical collar for at least 4~6 weeks after the operation, which may lead to no obvious changes during the follow-up period.

Further study is supposed to enlarge the sample size and prolong the follow-up period. What is more, in this study, only patients with cervical spondylosis who underwent single-segment ACDF were selected, and patients with multi-segment lesions need to be further studied. Besides, the ratio of 1.20 to 1.80 times distraction is obtained in this study. Even so, the relationship between the specific cage size and intervertebral distraction needs to be further studied, because the specific position of cage implantation and the curvature of cervical vertebra have a certain influence on the degree of distraction in addition to the obvious effect of cage size on intervertebral distraction. Consequently, in the future, we plan to collect large samples and analyze the specific relationship between the specific location of cage size, implant position, cervical curvature, and intervertebral distraction through utilizing big data.

## Conclusions

The cages implantation and intervertebral distraction would increase the area and height of the surgical segment foramen when performing ACDF. However, the oversize cage could have a negative impact on the area and height of the adjacent intervertebral foramen. Besides, the mean value of the adjacent intervertebral disk space height could be used as a reference standard. Furthermore, a cage with 1.20~1.80 fold distraction could achieve better long-term results. Apart from the above, ACDF could effectively restore the segmental lordosis of the cervical spine, but it barely affects the overall curvature, which might be caused by compensation of adjacent segments.

## Data Availability

The datasets generated and analyzed during the current study are available from the corresponding author on reasonable request.

## Abbreviations

ACDF: Anterior cervical discectomy and fusion
PEEK: Polyetheretherketone
JOA: Japanese Orthopaedic Association Scores
VAS: Visual analog scales
*H*: The surgical intervertebral space height after surgery
*H*_0_: The surgical intervertebral space height before surgery
*H*_1_: The upper segmental intervertebral space height before surgery
*H*_2_: The lower segmental intervertebral space height before surgery
*H*_m_: The preoperative mean height of adjacent segments, *H*_m_=(*H*_1_+*H*_2_)/2
Δ*H*: The reduction of intervertebral space height
ΔSH_1_: The reduction of intervertebral space height at the adjacent upper segment after surgery (ΔSH_1_=*H*_1_−postoperative *H*_1_)
ΔSH_2_: The reduction of intervertebral space height at the adjacent lower segment after surgery (ΔSH_2_=*H*_2_−postoperative *H*_2_)
ΔFH_1_: The reduction of intervertebral space height of the adjacent upper segment at the last follow-up (ΔFH_1_=*H*_1_−follow-up *H*)
ΔFH_2_: The reduction of intervertebral space height of the adjacent lower segment at the last follow-up (ΔFH_2_=*H*_2_−follow-up *H*_2_)
*α*_1_: Regional curvature (segmental lordosis)
*α*_2_: Global curvature (overall lordosis)
